# Plaque Rupture in a Hodgkin Lymphoma Survivor without Cardiovascular Risk Factors 20 Years after Thoracic Radiotherapy: A Case Report

**DOI:** 10.3390/jcdd10080324

**Published:** 2023-07-31

**Authors:** Elissa A. S. Polomski, Michiel A. de Graaf, J. Wouter Jukema, M. Louisa Antoni

**Affiliations:** Department of Cardiology, Heart Lung Center, Leiden University Medical Center, Albinusdreef 2, 2333 ZA Leiden, The Netherlands

**Keywords:** Hodgkin lymphoma, radiation-induced heart disease, acute myocardial infarction, cardio-oncology, case report

## Abstract

Background: Major improvements in cancer therapies have significantly contributed to increased survival rates of Hodgkin lymphoma (HL) survivors, outweighing cardiovascular side effects and the risks of radiation-induced heart disease. Non-invasive screening for coronary artery disease (CAD) starting five years after irradiation is recommended, as plaque development and morphology may differ in this high-risk population. Due to rapid plaque progression and a possibly higher incidence of non-calcified plaques, coronary artery calcium scoring may not be sufficient as a screening modality in HL survivors treated with thoracic radiotherapy. Case summary: A 42-year-old man with a history of HL treated with thoracic radiotherapy presented at the emergency department 20 years after cancer treatment with an ST-elevation myocardial infarction, in the absence of cardiovascular risk factors, for which primary percutaneous coronary intervention of the left anterior descending artery was performed. Four months prior to acute myocardial infarction, invasive coronary angiography only showed wall irregularities. Two years earlier, the Agatston calcium score was zero. Discussion: In HL survivors treated with thoracic radiotherapy, a calcium score of zero may not give the same warranty period for cardiac event-free survival compared to the general population. Coronary computed tomography angiography can be a proper diagnostic tool to detect CAD at an early stage after mediastinal irradiation, as performing calcium scoring may not be sufficient in this population to detect non-calcified plaques, which may show rapid progression and lead to acute coronary syndrome. Also, intensive lipid-lowering therapy should be considered in the presence of atherosclerosis in this patient population.

## 1. Introduction

Over the past decades, major improvements in cancer therapies have significantly contributed to increased survival rates, outweighing the cardiovascular side effects [1]. Almost 50% of cancers nowadays are eligible for treatment with radiotherapy, increasing the risk of radiation-induced heart disease (RIHD) [2]. The risk of coronary artery disease (CAD) in cancer survivors treated with thoracic radiotherapy increases at a younger age with radiation exposure, higher radiation doses, and the presence of pre-existing cardiovascular risk factors [3]. According to the most recent guidelines of the European Society of Cardiology (ESC) on cardio-oncology, prior mean heart doses of >25 Gray (Gy) or ≥35 Gy to a volume exposing the heart are associated with a very high cardiovascular toxicity risk. Therefore, the guidelines recommend starting non-invasive screening for CAD five years after irradiation, with intervals of 5–10 years [4].

In the pathophysiology of conventional atherosclerosis, endothelial injury is caused by cholesterol load, shear stress, and oxidative stress, changing macrophages into foam cells and maintaining a pro-inflammatory environment. The pathophysiological mechanism of RIHD is characterized by the inflammatory responses of macrophages, which change the phenotypes of all vessel wall layers, ultimately leading to intimal, and medial thickening due to extracellular matrix deposition and a state of chronic inflammation [5]. Long-term risks of (fatal) cardiovascular disease after irradiation have been described [6], but more extensive research should be performed on radiation-induced pathophysiology and the acceleration of atherosclerosis.

Cancer survivors treated with mediastinal irradiation more often present with ostial and proximal coronary lesions and more extensive CAD compared to the general population [7]. Additionally, plaque development and morphology may differ in these patients, and radiotherapy-induced atherosclerosis may lead to non-calcified morphologies more often, which cannot be detected by coronary artery calcium (CAC) scoring [8,9,10]. In this case report, we present a patient without cardiovascular risk factors and minimal atherosclerosis on invasive coronary angiography four months prior to acute myocardial infarction.

## 2. Case Presentation

A 42-year-old man presented to the emergency department of our hospital with complaints of burning chest pain radiating to the jaw. The electrocardiogram (ECG) showed elevated ST segments inferior and lateral. Invasive coronary angiography showed severe stenosis and thrombotic occlusion of the proximal left anterior descending artery (LAD). He underwent primary percutaneous coronary intervention (PCI) with the placement of one drug-eluting stent (DES).

**Figure 1:**
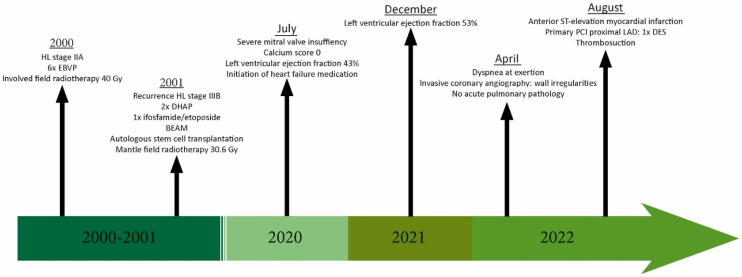
**Timeline**. HL = Hodgkin lymphoma; EBVP = Epirubicin, Bleomycin, Vincristin, Prednisolon; Gy = Gray, DHAP = Dexamethasone, High dose Ara-C, Platinol; BEAM = Carmustine, Etoposine, Cytarabine, Melphalan; PCI = percutaneous coronary intervention; LAD = left coronary descending artery; DES = drug-eluting stent.

Twenty-two years earlier, our patient was diagnosed with Hodgkin lymphoma (HL) stage IIA, localized in the mediastinal and right supraclavicular areas, followed by a recurrence of HL stage IIIB in the left axillary region, in the para-aortic and iliac lymph nodes, and spleen one year later. He was treated with chemotherapy (EBVP, DHAP, BEAM, and ifosfamide/etoposide), radiotherapy (involved field and mantle field), and autologous stem cell transplantation. Involved field radiation doses were given in fractions of 1.8 Gy and 2.0 Gy, with maximum doses of 30.6 Gy and 40 Gy, respectively. During mantle field therapy, our patient was exposed to a maximum radiation dose of 14 Gy. Unfortunately, information about the cumulative heart dose was not available. The chronological summary of visits is shown in the timeline in Figure 1.

**Figure 2:**
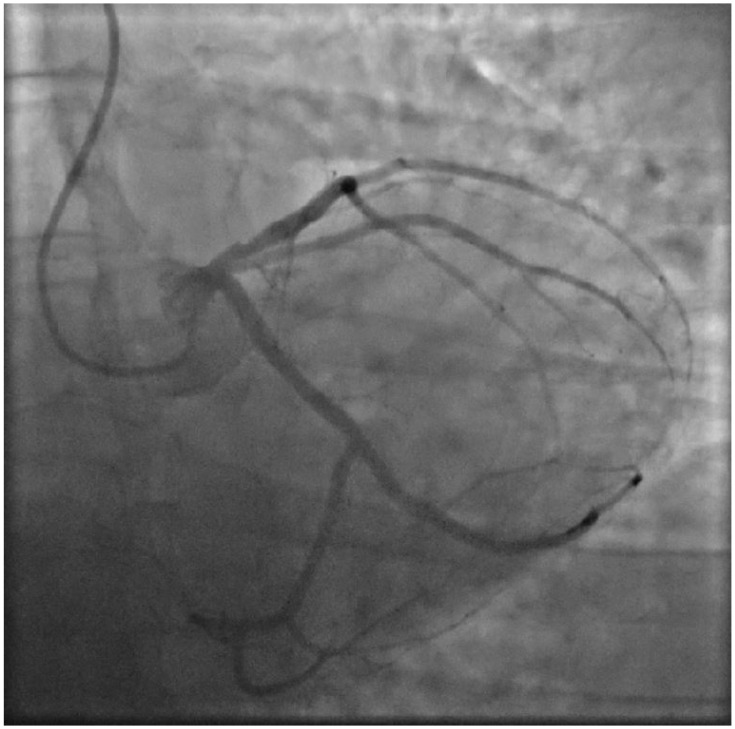
**Invasive coronary angiography**. The LAD during invasive coronary angiography in April 2022. Wall irregularities in the proximal LAD are seen.

Twenty years after cancer treatment, our patient visits the outpatient cardiology clinic with complaints of dyspnea. In the absence of any cardiovascular risk factors, transthoracic echocardiography in 2020 showed severe mitral valve insufficiency based on Carpentier type IIIb. He was diagnosed with heart failure (HF) with a left ventricular ejection fraction (LVEF) of 43% on cardiac magnetic resonance imaging (MRI), which improved to 53% in 2021 after the initiation of HF medication (bumetanide, losartan, spironolactone, bisoprolol, and ivabradine). Blood pressure and cholesterol levels were well regulated (total cholesterol 3.38 mmol/L) and our patient had no significant history of smoking (0.75 pack years). Their family history was negative for CAD. A CAC scan was performed, and the calculated Agatston score was zero. In the spring of 2022, our patient presented at the outpatient cardiology clinic with complaints of progressive dyspnea at exertion. Transesophageal and transthoracic echocardiography showed good left and right ventricular function and moderate mitral insufficiency. Invasive coronary angiography showed wall irregularities without any significant obstructions (Figure 2), and statins were not prescribed. Except for known para mediastinal bronchopathy, the chest X-ray did not show any abnormalities. Possible differential diagnoses included interstitial lung disease and deconditioning. Due to persistent complaints, he was referred to the outpatient cardio-oncology clinic of our center. According to the New York Heart Association Functional Classification, based on patient symptoms, our patient was graded as Class III. Physical examination showed no signs of congestion, and during exercise stress testing, a moderate functional capacity of 160 watts (79%), a maximum oxygen uptake of 17.70 mL/kg (71%), and a respiratory exchange ratio of 1.03 was observed. Transthoracic echocardiography showed grade II diastolic dysfunction, good right and left ventricular function (LVEF 55%), and mild mitral regurgitation.

**Figure 3:**
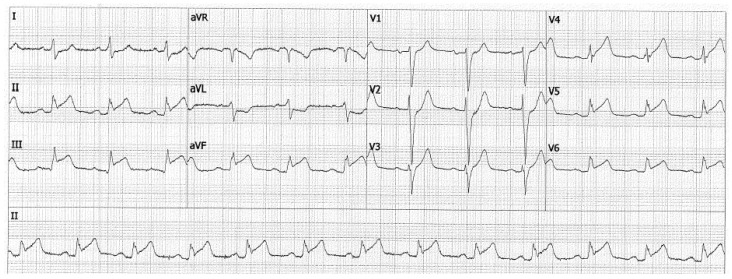
**Electrocardiogram.** The patient’s ECG during a presentation at the emergency department of our center.

**Figure 4:**
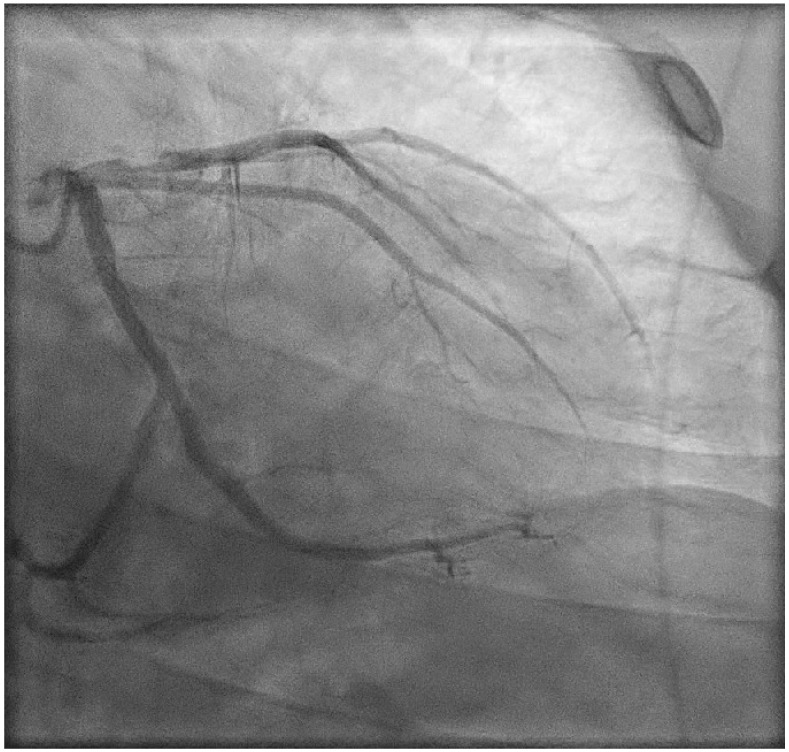
**Invasive coronary angiography.** The LAD during primary PCI after our patient presented with acute myocardial infarction. Significant stenosis of the proximal LAD is shown.

Three months later, our patient presented in a hemodynamically stable condition at the emergency department with an ST-elevation myocardial infarction (STEMI) for which primary PCI with placement of one DES in the proximal LAD was performed (Figure 3 and Figure 4). At the presentation, there was no evidence of heart failure (Kilip Class I). After the procedure, TIMI flow in the LAD was 2–3: the proximal and mid part of the LAD and the diagonal showed normal flow, in the distal/apical part of the LAD the flow was reduced. Our patient was treated with Aggrastat. Left ventricular function was not impaired. Clopidogrel was prescribed for one year, as the patient was a normal metabolizer of CYP2C19 which is routinely tested in our hospital, and there is a life-long indication for acetylsalicylic acid. He was referred to the vascular medicine department, where coagulation disorders as the underlying cause of STEMI were ruled out.

## 3. Discussion

This patient case shows a rupture of a non-significant soft plaque in an HL survivor treated with thoracic radiotherapy without cardiovascular risk factors. Our patient had a CAC score of zero in 2020 but presented with an acute cardiovascular event only two years later. In the general population, a CAC score of zero is associated with a very low risk of a cardiovascular event in the following five years [11]. However, this may be different for cancer survivors treated with thoracic radiotherapy, and, therefore, performing CAC scoring for risk estimation may not be sufficient in this specific population [12,13].

### 3.1. Concomitant Anthracycline Chemotherapy

The incidence and progression of radiation-related cardiovascular complications depend on the dose of radiation, concomitant cancer therapies, and patient characteristics such as pre-existing cardiovascular disease, risk factors, and age. Our patient was treated with anthracycline-containing chemotherapeutic regimens in addition to radiotherapy. Several studies have observed an increased risk of cardiotoxicity after treatment with anthracyclines [14,15]. However, a study by Hancock et al. described a similarly elevated risk of cardiac disease for HL survivors treated with radiotherapy alone or in combination with chemotherapy [16]. Although anthracycline therapy does not increase the excess relative risk of developing cardiac disease per Gy of radiation compared to patients not treated with anthracyclines, it does accelerate the onset of cardiac diseases including myocardial infarction, angina pectoris, and HF. A large study on childhood cancer survivors reported that five-year childhood cancer survivors who were treated with >15 Gy heart dose radiation combined with anthracyclines had an earlier onset of cardiac disease [16,17].

### 3.2. Non-Invasive Modalities for Detecting CAD

At this moment, invasive coronary angiography is the gold standard for the detection of CAD. Nevertheless, a large study on HL survivors showed that coronary computed tomography angiography (CCTA) can be a proper diagnostic tool to detect CAD at an early stage after mediastinal irradiation [9].

Various other imaging modalities are used in clinical practice to assess CAD or perfusion abnormalities. Stress echocardiography, nuclear imaging, cardiac magnetic resonance imaging, and CCTA all have strengths and weaknesses in their sensitivity, specificity, and overall ability to detect and quantify cardiac damage. Stress echocardiography (exercise or dobutamine) has low cost, minimal risk, and no exposure to ionizing radiation. It can recognize structural and functional alterations not evident at rest; this method is highly sensitive and specific for detecting abnormalities in the epicardial coronary arteries. It is frequently used to evaluate patients for myocardial ischemia. The accuracy of stress echocardiography is dependent on the acoustic window and the experience of the operator. Previous studies have reported a positive predictive value of 80% to detect severe three-vessel disease and 87% for left main CAD after Hodgkin disease [18].

ECG-gated myocardial perfusion single-photon emission computed tomography (SPECT) with the myocardial perfusion tracers ^99m^Tc-sestamibi or ^99m^Tc-tetrofosmin can be used to assess radiation-induced myocardial perfusion defects, indicative of vascular damage or ischemia. Reversible or irreversible perfusion defects are marked by decreased tracer uptake [19]. Various studies have described short-term as well as long-term perfusion defects after irradiation, mainly in breast cancer survivors, and found an association between the area of the heart that had been exposed to radiotherapy and the location of perfusion defects, as well as dose-dependent relationships for regional perfusion defects [20,21,22]. Studies also observed a linear association between the percentage of the left ventricle that was present in the radiation field and the number of patients with perfusion defects [23,24,25,26]. However, heart-sparing radiation techniques were not widely implemented yet, and future studies on irradiated patients with heart-sparing techniques did not observe perfusion defects post-irradiation [27,28]. More recently, positron emission tomography (PET) scanning using different tracers, including ^82^Rb, emerged as a non-invasive modality to assess myocardial blood flow and perfusion defects [29]. Measuring the myocardial blood flow using ^82^Rb can detect myocardial ischemia and microvascular CAD, which are of prognostic value for future cardiac events [30]. PET tracers have a more linear relationship with the myocardial blood flow and therefore a higher sensitivity and specificity for significant stenosis compared to the abovementioned SPECT tracers [31,32,33,34]. Also, PET scans are less time-consuming than SPECT and expose the patient to a lower radiation dose [32]. Moreover, as ^82^Rb is reported to be an independent predictor of three-vessel disease, in patients with suspected or known CAD, PET should be preferred above SPECT, as SPECT can miss balanced three-vessel disease [35].

Myocardial perfusion MRI uses chelated gadolinium contrast to evaluate rest and stress perfusion and observe hypo-enhancement, which correlates with microvascular damage. The prognostic value, sensitivity, and specificity of MRI exceed those of SPECT and in low-intermediate-risk patients, a negative stress MRI decreases the risk of CAD, while a positive stress MRI increases the risk of CAD in intermediate-high-risk patients. However, false-positive results are also reported, caused by dark-rim artifacts that hamper the interpretation of myocardial perfusion MRI [36,37]. Although MRI informs about the area of the perfusion defects and the location of obstructive stenosis can be estimated, this modality does not give any information about the severity of stenosis, plaque morphology, or high-risk plaque features, equivalent to SPECT and PET [38]. Compared to myocardial perfusion modalities, CCTA provides detailed anatomical information on the coronary arteries and can identify the location of lumen narrowing accurately, as well as high-risk plaque features.

The most recent guidelines by the Society of Cardiovascular Computed Tomography also recommend determining the atherosclerosis-related cardiovascular risk score in cancer survivors to guide the initiation of treatment and screening with CAC scans or available non-gated non-contrast CT scans [39]. Although the cardio-oncology guidelines by the ESC recommend starting screening for CAD in asymptomatic cancer survivors five years after cancer treatment using a non-invasive screening modality, the preferred modality is not specified [4]. This patient case shows that performing calcium scoring may not be sufficient in a population of irradiated HL patients, as non-calcified plaque morphologies are not detected, and that a calcium score of zero may not give the same warranty period for cardiac event-free survival compared to the general population. In this patient case, perfusion imaging would have been normal because of the non-significant plaque; however, a CCTA scan as a screening method would have given more information on plaque characteristics, which were now undetected by coronary angiography unless intravascular ultrasound (IVUS) or optical coherence tomography (OCT) were performed additionally.

Pathological studies have demonstrated that plaques with a thin-cap fibroatheroma phenotype, including a thin fibrous cap, large lipid pool, necrotic core, microcalcifications, cholesterol crystals, inflammatory infiltrates, including activated macrophages near the fibrous cap, and intraplaque hemorrhage, underscore 55–60% of plaque rupture episodes. Using IVUS, minimum lumen area ≤ 4 mm^2^, a plaque burden ≥ 70%, and the virtual histology thin cap fibroatheroma phenotype were more likely to cause cardiovascular events than simple lesions. More recently, OCT has been increasingly used in intravascular coronary imaging. Similarly to IVUS, OCT provides cross-sectional images of the vessel. However, instead of ultrasound, OCT employs light for tissue analysis, which enables visualization of coronary lesions with almost microscopic precision. The spatial resolution of OCT is approximately 10 times higher than that of IVUS to visualize plaque features, but OCT lacks the penetration depth to image the entire plaque burden. OCT determined lipid-rich plaques with macrophage infiltrates as high-risk features for cardiovascular events [40].

Further research is needed to determine which modality should be implemented as the preferred modality for RIHD.

## 4. Conclusions

The progression and morphology of coronary atherosclerotic lesions may be different in HL survivors treated with thoracic radiotherapy. This patient case illustrates the importance of screening these patients for CAD with a modality that detects (high-risk) plaque features and morphology in order to start intensive lipid-lowering therapy in the presence of minimal atherosclerosis.

## Data Availability

No new data were created or analyzed in this study. Data sharing is not applicable to this article.
